# Incidence of infection following internal fixation of open and closed tibia fractures in India (INFINITI): a multi-centre observational cohort study

**DOI:** 10.1186/s12891-017-1506-4

**Published:** 2017-04-14

**Authors:** Prakash Doshi, Hitesh Gopalan, Sheila Sprague, Chetan Pradhan, Sunil Kulkarni, Mohit Bhandari

**Affiliations:** Division of Orthopaedic Surgery, McMaster University, Well-Health Building, 293 Wellington Street North, Suite 110, Hamilton, ON L8L 8E7 Canada

**Keywords:** Closed tibia fractures, Open tibia fractures, Wound infection

## Abstract

**Background:**

Trauma is a major public health problem, particularly in India due to the country’s rapid urbanization. Tibia fractures are a common and often complicated injury that is at risk of infection following surgical fixation. The primary objectives of this cohort study were to determine the incidence of infection within one year of surgery and to describe the distribution of infections by location and time of diagnosis for tibia fractures in India.

**Methods:**

We conducted a multi-center, prospective cohort study. Patients who presented with an open or closed tibia fracture treated with internal fixation to one of the participating hospitals in India were invited to participate in the study. Participants attended follow-up visits at 3, 6, and 12 months post-surgery, where they were assessed for infections, fracture healing, and health-related quality of life as measured by the EurQol-5 Dimensions (EQ-5D).

**Results:**

Seven hundred eighty-seven participants were included in the study and 768 participants completed the 12 month follow-up. The overall incidence of infection was 2.9% (23 infections). The incidence of infection was 1.6% (10 infections) in closed and 8.0% (13 infections) in open fractures. There were 7 deep and 16 superficial infections, with 5 being early, 7 being delayed, and 11 being late infections. Intra-operative antibiotics were given to 92.1% of participants and post-operative antibiotics were given to 96.8% of participants. Antibiotics were prescribed for an average of 8.3 days for closed fractures and 9.1 days for open fractures. Infected fractures took significantly longer to heal, and participants who had an infection had significantly lower EQ-5D scores.

**Conclusions:**

The incidence of infection within this cohort is similar to those seen in developed countries. The duration of prophylactic antibiotic use was longer than standard practice in North America, raising concern for the potential development of antibiotic resistant microbes within Indian orthopaedic settings. Future research should aim to identify the best practice for antibiotic use in India to ensure that antibiotic usage patterns do not lead to unnecessary overuse, while maintaining a low incidence of infection.

**Trial registration:**

NCT01691599, September 17, 2012.

## Background

Trauma is a major public health problem in India as a result of accelerated urbanization and industrialization [1]. The increase in trauma in recent years has led to a greater incidence of fractures treated with internal fixation, with tibia fractures being reported as one of the most common and complicated fractures [1, 2]. Tibia fractures that are treated with surgery are at risk of serious and debilitating infections [3]. Previous research has suggested that the incidence of infections following internal fixation is higher in low and middle income countries (LMICs), as operating rooms are often not sterile and may contain microbes responsible for wound infection [4–6].

Surgical site infection (SSI) following internal fracture fixation poses large socioeconomic and quality of life implications for the patient [3, 7]. There is an increased risk within LMICs due to the disproportionately large number of trauma incidents, particularly attributed to motor vehicle accidents, coupled with the aforementioned risk factors of poor sterilization of orthopaedic wards and long times between fracture and surgery [4–6].

The primary objectives of the current study were to determine the incidence of infection within one year of surgery and to describe the distribution of infections by location (superficial, deep) and time of diagnosis (early, delayed, late) for open and closed tibia fracture patients in India. Secondary objectives were to: 1) describe the symptoms, management, and treatment outcomes of infections, 2) explore the effect of fracture type, hospital type, time to surgery, and planned duration of antibiotics on the incidence of infection, 3) compare the proportion of fractures healed at 12 months in patients with and without infections, and 4) evaluate health-related quality of life over 12 months in patients with and without infections.

## Methods

### Study overview

We conducted a multi-center, prospective, observational cohort study to investigate the incidence of infections within one year for open and closed tibia fracture patients who were treated with internal fixation. The method of internal fixation was left to the discretion of the attending surgeon. After obtaining informed consent, baseline and surgical data were recorded. Approval was obtained from the Institutional Review Board (Aurora, Ontario) and each hospital’s local Ethics Committee prior to commencing study activities.

### Participant identification and eligibility criteria

Patients who presented to one of the participating hospitals with a tibia fracture treated with internal fixation were screened for study eligibility. The inclusion criteria were: 1) Men and women who are 18 years of age or older. 2) Open or closed tibia fracture (AO 41, 42, and 43) treated by internal fixation (plate or nail) or by external fixation with planned conversion to plate or nail. 3) Ability to understand the content of the subject information/informed consent form and to be willing to participate in the clinical investigation. 4) Provided written informed consent.

The exclusion criteria were: 1) Previous wound infection or osteomyelitis at the same limb (according to subject history). 2) Patients who plan to undergo conversion surgery at a different hospital. 3) Previous fracture with retained hardware in injured extremity that will interfere with implant fixation. 4) Immunological deficiency disease. 5) Tumor related fractures. 6) Any severe systemic disease: class V-VI of the American Society of Anesthesiologists (ASA) physical status classification [8]. 6) Recent history of substance abuse that would preclude reliable assessment. 7) Patient is a prisoner. 8) Participation in any other medical device or medicinal product study within the previous month that could influence the results of the present study. Reasons for ineligibility were documented.

### Data collection

After providing informed consent, baseline information was documented and participants underwent a haematology analysis (Leucocyte count, CRP level, and ESR level) and radiographs (AP, lateral) before surgery. Details regarding the surgical procedure, including antibiotic prophylaxis, were documented. Post-operatively participants underwent a haematology analysis and x-rays.

Participants with any symptoms of surgical site infections underwent further investigations including radiological assessment, hematological analysis and bacteriological culture and antibiogram whenever possible to determine whether infection was present. If infection was diagnosed, infection management including administered antibiotics, wound care, surgical intervention performed, and infection treatment outcome were recorded.

Participants attended clinic visits at 3 months, 6 months, and 12 months post-surgery, or were contacted by telephone to collect as much information as possible if unable to attend a follow-up visit. At each visit, patients were assessed for infections and fracture healing. Haematology and radiographs were taken as standard of care. Antibiotic use was documented. Participants also completed the EuroQol-5 Dimensions (EQ-5D). The EQ-5D is a standardized instrument for use as a measure of health outcome, primarily designed for self-completion. At the 12 month visit, any planned revision surgeries were also documented.

### Confirmation of eligibility and review of infections

An independent Adjudication Committee comprised of three orthopaedic trauma surgeons confirmed the eligibility for cases in which patient eligibility was in doubt. They also reviewed reported infections to confirm the presence of infection and classify the infection as a superficial incisional surgical site infection (SSI) or a deep incisional SSI using CDC criteria [9]. They also confirmed the timing of infection as early (onset of symptoms within 2 weeks of injury), delayed (onset of symptoms 2–10 weeks after injury) or, late (onset of symptoms more than 10 weeks after injury).

### Data analysis

We summarize participant characteristics using descriptive statistics expressed as means and standard deviations for continuous variables) or counts and percentages for categorical variables. For analysis of primary outcomes, the incidence of infection within one year of the internal fixation surgery was reported as a proportion. A Fisher’s exact test was used to determine if the incidence of infection and infection type (superficial versus deep) differed across fracture types (open versus closed fractures). Infections were classified by timing of onset and a Chi-square test was used to compare the incidence of early versus delayed versus late onset infections across fracture types.

For analysis of secondary outcomes, infection symptoms and management in open and closed fracture patients were summarized using descriptive statistics. Infection outcomes and fracture healing at 12 months for open and closed fracture patients are reported as proportions. A Chi-square test was used to compare if the incidence of infections differed by hospital type (public versus private versus combination), as well as by fixation technique used and Gustilo classification for open fractures. Fisher’s exact tests were used to determine if the incidence of infection differed across timing of surgery (within 6 h of injury versus greater than 6 h from injury), and by fixation device material (stainless steel versus titanium). Fracture healing status at 12 months in patients with an infection versus those without was also compared using a Fischer’s exact test. The EQ-5D scores are reported as means and standard deviations for participants with and without infections. Time to healing in patients with and without infections was explored using a *t*-test, and EQ-5D scores in patients with and without infections were compared using two-way repeated measures ANOVA. Level of significance was determined as *p* < 0.05. Data analysis was done using Statistical Analysis Software (SAS, v9.2, Cary, North Carolina, United States).

## Results

**Fig. 1:**
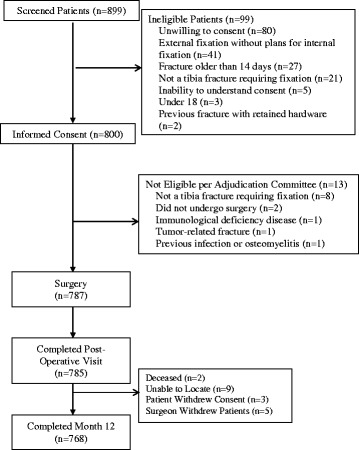
Participant Flow Diagram

Of the 899 patients screened for participation, 800 met the inclusion criteria and provided informed consent (Fig. 1). The Adjudication Committee deemed 13 participants to be ineligible. 787 participants were included in the analyses and 768 participants completed the 12 month follow-up.

### Demographics and fracture characteristics

The mean age of the study participants was 40.1 ± 14.0 years and the majority were male (79.8%) (Table 1). The majority of participants included in this study were healthy (88.9% had no comorbidities) and non-smokers (95.6%). Less than 20% of participants had insurance. Most participants had completed secondary school (33.8%), junior college (14.2%), or university (36.0%). The most common mechanism of injury was motor vehicle accidents (71.4%) (Table 2). Less than one-third of participants (28.5%) had additional injuries or fractures. The majority of participants had closed fractures (625 participants, 79.4%), with 162 participants (20.6%) having open fractures.

**Table 1:** Participant Demographics

Characteristic	N (%) *N* = 787
Age (mean ± SD)	40.1 ± 14.0
Sex	
Female	159 (20.2%)
Male	628 (79.8%)
Ethnicity	
Indian	787 (100.0%)
Education	
None	52 (6.6%)
Primary school	74 (9.4%)
Secondary school	266 (33.8%)
Junior college	112 (14.2%)
University	283 (36.0%)
Insurance	
None	650 (82.6%)
Governmental	27 (3.4%)
Private	110 (14.0%)
Smoker	
No	752 (95.6%)
Yes	22 (2.8%)
Former	13 (1.7%)
Co-morbidity	
None	700 (88.9%)
Yes^a^	87 (11.1%)
High Blood Pressure	54 (62.1%)
Heart Disease	16 (18.4%)
Osteoporosis	5 (5.7%)
Osteoarthritis or	5 (5.7%)
Degenerative Arthritis	
Lung Disease	4 (4.6%)
Stomach Disease or	3 (3.4%)
Ulcer	
Kidney Disease	2 (2.3%)
Liver Disease	2 (2.3%)
Hepatitis B	2 (2.3%)
Epilepsy	2 (2.3%)
Blood Disorder or	1 (1.1%)
Anemia	
Osteopenia	1 (1.1%)
Cancer	1 (1.1%)
Cervical Tubercular	1 (1.1%)
Lymphadenitis	
Fever Since 2 Days	1 (1.1%)
Cough	1 (1.1%)
Hyperlipidemia	1 (1.1%)
Unspecified	1 (1.1%)
Diabetic	
No	734 (93.3%)
Yes – Insulin-dependent	24 (3.0%)
Yes – Insulin-independent	29 (3.7%)

^a^Does not equal to 100% due to patients having multiple comorbidities

**Table 2:** Injury and Fracture Characteristics

Characteristic	N (%) *N* = 787
Mechanism of injury	
Motor vehicle accident	562 (71.4%)
Fall	186 (23.6%)
Struck	30 (3.8%)
Other	3 (0.4%)
Twisting	3 (0.4%)
Sports	2 (0.3%)
Stress Fracture	1 (0.1%)
Work-related injury	70 (8.9%)
Additional fractures / injuries	224 (28.5%)
AO – Müller Fracture Classification	
41-Proximal	255 (32.4%)
42-Diaphyseal	337 (42.8%)
43-Distal	95 (12.1%)
44-Malleolar	100 (12.7%)
Open fracture	162 (20.6%)
Gustilo classification	
I	67 (41.4%)
II	44 (27.2%)
IIIA	28 (17.3%)
IIIB	21 (13.0%)
IIIC	1 (0.6%)
Closed fracture	625 (79.4%)
Tscherne classification	
0	276 (44.2%)
1	251 (40.2%)
2	82 (13.1%)
3	16 (2.6%)

Totals may not add due to missing data

### Surgical management and peri-operative care

The mean time from injury to surgery was 73 ± 107.5 h (Table 3). The time to surgery was 64 ± 108.0 h for open fractures and 76 ± 108.0 h for closed fractures. The majority of fractures were stabilized with a plate (43.2%) or a reamed intramedullary nail (41.3%). The majority of implants were from local (Indian) manufacturers (86.0%) versus global manufacturers (14.0%).

**Table 3:** Surgical and Peri-Operative Management

	Open *N* = 162 N (%)	Closed *N* = 625 N (%)	Total *N* = 787 N (%)
Duration time from injury to surgery (hours) (Mean ± SD)	64.0 ± 108.0	76.0 ± 108.0	73.1 ± 107.5
Duration time from hospital admission to surgery (hours) (Mean ± SD)	55.1 ± 78.0	59.0 ± 78.0	58.1 ± 77.3
Previous irrigation and debridement	57 (35.2%)	1 (0.2%)	58 (6.5%)
Duration of definitive fixation surgery (hours) (Mean ± SD)	2.12 ± 1.0	1.53 ± 1.0	1.57 ± 1.0
Method of fixation			
Plate	32 (19.8%)	308 (49.3%)	340 (43.2%)
Reamed intramedullary nail	102 (63.0%)	223 (35.7%)	325 (41.3%)
Screw	7 (4.3%)	62 (9.9%)	69 (8.8%)
Unreamed intramedullary nail	13 (8.0%)	16 (2.6%)	29 (3.7%)
Screw and wire	4 (2.5%)	5 (0.8%)	9 (1.1%)
Wire	0 (0.0%)	5 (0.8%)	5 (0.6%)
Intramedullary nail (unspecified)	3 (1.9%)	1 (0.2%)	4 (0.5%)
Plate and wire	0 (0.0%)	2 (0.3%)	2 (0.3%)
Plate and reamed nail	0 (0.0%)	1 (0.2%)	1 (0.1%)
External fixator	0 (0.0%)	1 (0.2%)	1 (0.1%)
Implant manufacturer			
Local	147 (90.7%)	530 (84.8%)	677 (86.0%)
Global	15 (9.3%)	95 (15.2%)	110 (14.0%)
Additional surgical procedures performed	43 (26.5%)	89 (14.2%)	132 (16.8%)
Antibiotics administered during surgery	149 (92.0%)	576 (92.2%)	725 (92.1%)
Participant returned to operating room for repeat debridement/irrigation	9 (5.6%)	1 (0.2%)	10 (1.3%)
Antibiotics received post-operative	157 (97.5%)	605 (97.0%)	762 (96.8%)
Planned duration of post-op antibiotics	9.1 days ± 5.0	8.3 days ± 5.0	8.5 days ± 5.0
Drains used post-op	110 (68.3%)	354 (56.7%)	464 (59.11%)
Wound manually cleaned	152 (94.4%)	415 (66.5%)	567 (72.3%)

Totals may not add due to missing data

Almost all participants with an open fracture had an irrigation and debridement (92.0%). Approximately one third (35.2%) of open fractures had an irrigation and debridement prior to their surgery for definitive fixation. Very few open fracture participants (5.6%) returned to the operating room for subsequent irrigation and debridement (Table 3). Post-operative drains were used in 68.3% of open fracture patients and 56.7% of closed fracture patients.

Almost all participants received antibiotics during surgery (92.1%) and after surgery (96.8%). The antibiotics were prescribed for an average of 9.1 ± 5.0 days in open fractures and 8.3 ± 5.0 days in closed fractures. The vast majority of patients received Cephalosporin (757 patients). The majority of open fracture participants (94.4%) had their wound cleaned manually post-operatively and two thirds of closed fracture participants (66.5%) had their wound cleaned manually (Table 3). Proximal fractures were most commonly treated with plating (87.8%), while diaphyseal fractures and distal fractures were most commonly treated with reamed intramedullary nailing (81.3%, and 49.5% respectively) (Table 4).

**Table 4:** Method of Fixation for AO Fracture Types

	AO Classification				Total *N* = 787 N (%)
	Proximal (*N* = 254)	Diaphyseal (*N* = 337)	Distal (*N* = 95)	Malleolar (*N* = 100)	
Method of fixation					
Plate	223 (87.8%)	36 (10.7%)	39 (41.0%)	42 (42.0%)	340 (43.2%)
Reamed intramedullary nail	2 (0.8%)	274 (81.3%)	47 (49.5%)	2 (2.0%)	325 (41.3%)
Screw	27 (10.6%)	1 (0.3%)	2 (2.1%)	39 (39.0%)	69 (8.8%)
Unreamed intramedullary nail	1 (0.4%)	22 (6.5%)	3 (3.2%)	3 (3.0%)	29 (3.7%)
Screw and wire	0 (0.0%)	0 (0.0%)	0 (0.0%)	9 (9.0%)	9 (1.1%)
Wire	0 (0.0%)	0 (0.0%)	2 (2.1%)	3 (3.0%)	5 (0.6%)
Intramedullary nail (unspecified)	0 (0.0%)	3 (0.9%)	1 (1.1%)	0 (0.0%)	4 (0.5%)
Plate and wire	0 (0.0%)	0 (0.0%)	0 (0.0%)	2 (2.0%)	2 (0.3%)
Plate and reamed nail	0 (0.0%)	1 (0.3%)	0 (0.0%)	0 (0.0%)	1 (0.1%)
External fixator	1 (0.4%)	0 (0.0%)	0 (0.0%)	0 (0.0%)	1 (0.1%)

Totals may not add due to missing data

### Incidence of infection

The incidence of infection within 12 months of surgery was 2.9% (23 participants). The incidence of infection was higher in open fractures (8.0%) (13 infections) as compared to closed fractures (1.6%) (10 infections) (*p* < 0.0001) (Table 5). Of the 13 infections in open fractures, 1 occurred in a Gustilo Type I fracture, 3 in Type II fractures, and 9 in Type III fractures (*p* = 0.0002) (Table 5). There were 7 deep infections (5 in open fractures and 2 in closed fractures) and 16 superficial infections (8 in open fractures and 8 in closed fractures) (*p* = 0.2362). There were 5 early infections, 7 delayed infections, and 11 late infections (*p* = 0.1675). Infections were seen within patients treated with plating (11 infections), reamed intramedullary nailing (7 infections), unreamed intramedullary nailing (3 infections), screw (1 infection), and unspecified intramedullary nailing (1 infection) (*p* = 0.1110). There was no significant difference between infection rate and implant material used (stainless steel versus titanium, *p* = 0.9643) (Table 5).

**Table 5:** Characteristics of Infected and Non-Infected Fractures

	Infection N(%) *N* = 23	No Infection N(%) *N* = 764	Total N(%) *N* = 787	*p* value
Fracture type				<0.0001
Open	13 (56.5%)	149 (19.5%)	162 (20.6%)	
Closed	10 (43.5%)	615 (80.5%)	625 (79.4%)	
Open fracture Gustilo classification (*N* = 162)				0.0002
I	1 (7.7%)	66 (44.3%)	67 (41.4%)	
II	3 (23.1%)	41 (27.5%)	44 (27.2%)	
IIIA	2 (15.4%)	26 (17.4%)	28 (17.3%)	
IIIB	7 (53.8%)	14 (9.4%)	21 (13.0%)	
IIIC	0 (0.0%)	1 (0.7%)	1 (0.6%)	
Closed Fracture Tscherne classification (*N* = 625)				0.0588
0	1 (10.0%)	275 (44.7%)	276 (44.2%)	
1	5 (50.0%)	246 (40.0%)	251 (40.2%)	
2	3 (30.0%)	79 (12.8%)	82 (13.1%)	
3	1 (10.0%)	15 (2.4%)	16 (2.6%)	
Hospital Type				0.9938
Private	20 (87.0%)	662 (86.6%)	682 (86.7%)	
Public	2 (8.7%)	65 (8.5%)	67 (8.5%)	
Combination	1 (4.3%)	37 (4.8%)	38 (4.8%)	
Surgical Delay >6 h				0.6921
Yes	21 (91.3%)	670 (87.8%)	691 (87.9%)	
No	2 (8.7%)	93 (12.2%)	95 (12.1%)	
Method of fixation				0.1110
Plate	11 (47.8%)	329 (43.1%)	340 (43.2%)	
Reamed intramedullary nail	7 (30.4%)	318 (41.6%)	325 (41.3%)	
Screw	1 (4.3%)	68 (8.9%)	69 (8.8%)	
Unreamed intramedullary nail	3 (13.0%)	26 (3.4%)	29 (3.7%)	
Screw and wire	0 (0.0%)	9 (1.2%)	9 (1.1%)	
Wire	0 (0.0%)	5 (0.7%)	5 (0.6%)	
Intramedullary nail (unspecified)	1 (4.3%)	3 (0.4%)	4 (0.5%)	
Plate and wire	0 (0.0%)	2 (0.3%)	2 (0.3%)	
Plate and reamed nail	0 (0.0%)	1 (0.1%)	1 (0.1%)	
External fixator	0 (0.0%)	1 (0.1%)	1 (0.1%)	
Implant Material				0.9643
Stainless steel	17 (73.9%)	567 (74.2%)	584 (74.2%)	
Titanium	6 (26.1%)	196 (25.7%)	202 (25.7%)	
Fracture healed radiographically by 12 months	13 (56.5%)	674 (88.2%)	687 (87.3%)	<0.0001
Radiographic healing time in days (Mean ± SD)	223.0 ± 102.6	149.2 ± 72.0	150.6 ± 73.3	0.0234

Totals may not add due to missing data

Infection incidence was 2.9% at both private hospitals and public or combination hospitals (*p* = 0.9938) (Table 5). There was no difference in the incidence of infection between participants who had surgery within 6 h of their injury compared to greater than 6 h of their injury (*p* = 0.6921) (Table 5).

### Fracture healing and health-related quality of life

**Fig. 2:**
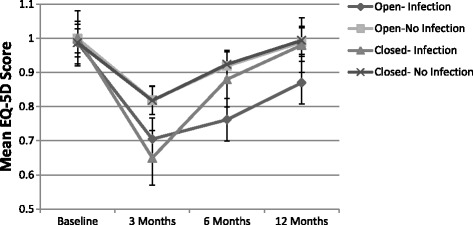
EQ-5D over time. Open-No Infection coincides with Closed-No Infection. Mean EQ-5D was used as a measure of health outcome over 12 months post-surgery

The mean time to fracture healing was 171.5 ± 88.4 days for open fractures and the majority (83.5%) had healed by 12 months. The mean fracture healing time for closed fractures was 145.5 ± 68.2 days and 91.0% had healed by 12 months. Fractures that were infected took 223.0 ± 102.6 days to radiographically heal, whereas fractures that were not infected took 149.0 ± 72.0 days to heal (*p* = 0.0234) (Table 5). Approximately half (56.5%) of the infected fractures were radiographically healed at 12 months, compared to almost all non-infected fractures (88.2%) (*p* < 0.0001) (Table 5). Participants who had an open fracture and an infection had the lowest EQ-5D scores at 6 months and 12 months (*p* < 0.0001) and their scores did not return to baseline at 12 months (Fig. 2).

### Infection characteristics and management

Symptoms of infections included purulent drainage (62.5%), wound healing disturbance (54.2%), erythema (37.5%), and local pain (33.3%) (Table 6). Bacterial cultures (52.2%) were taken in approximately half of the participants with infections and positive bacterial cultures were found in 44.4% of the cultures taken. The most common classification of organisms isolated was aerobic gram positive (69.2%) and aerobic gram negative (15.4%). The most commonly isolated organism was staphylocococcus aureaus (84.6%).

**Table 6:** Infection Symptoms and Management

	Open N(%) *N* = 13	Closed N(%) *N* = 10	Total N(%) *N* = 23
Symptoms present			
Purulent drainage	7 (53.8%)	7 (70.0%)	14 (60.9%)
Wound healing disturbance	5 (38.4%)	7 (70.0%)	12 (52.2%)
Persisting/increasing local pain	6 (46.2%)	1 (10.0%)	7 (30.4%)
Erythema	3 (23.1%)	5 (50.0%)	8 (34.8%)
Edema	1 (7.7%)	2 (20.0%)	3 (13.0%)
Fever	2 (15.4%)	0 (0.0%)	2 (8.3%)
Pain	1 (7.7%)	0 (0.0%)	1 (4.2%)
Maggots	1 (7.7%)	0 (0.0%)	1 (4.2%)
Bacteria culture found positive	5 (71.4%)	5 (83.3%)	10 (76.9%)
Class of organisms isolated			
Aerobic Gram positive	4 (80.0%)	3 (60.0%)	7 (70.0%)
Aerobic Gram negative	1 (20.0%)	0 (0.0%)	1 (10.0%)
Anaerobic Gram positive	0 (0.0%)	1 (20.0%)	1 (10.0%)
Anaerobic Gram negative	0 (0.0%)	1 (20.0%)	1 (10.0%)
Organisms collected			
*Staphylococcus aureus*	4 (80.0%)	5 (83.3%)	9 (81.8%)
*Pseudomonas* spp.	1 (20.0%)	0 (0.0%)	1 (9.1%)
*Enterobacter Serus*	0 (0.0%)	1 (16.7%)	1 (9.1%)
Infection treated by			
Antibiotics only	7 (53.8%)	6 (60.0%)	13 (56.5%)
Antibiotics and Surgery	6 (46.2%)	2 (20.0%)	8 (34.8%)
Surgery only	0 (0.0%)	2 (20.0%)	2 (8.7%)
Drainage used	4 (30.8%)	5 (50.0%)	9 (39.1%)
Wound manually cleaned	12 (92.3%)	10 (100.0%)	22 (95.6%)

Totals may not add due to missing data

The majority of the infections were treated with antibiotics only (53.8%) or with antibiotics and surgery (38.5%). Cephalosporin was the most commonly used antibiotic. Drainage was used in 30.8% of participants with infections and open fractures and 50.0% of participants with closed fractures and infections, and the wound was manually cleaned in 96.2% of participants. At 12 months, 4 infections had completely resolved without persistent drainage and recovery was still in progress for 19 infections.

## Discussion

This study found a low incidence of infections following surgical management of tibia fractures in a cohort of 787 participants in India. The incidence of infection for closed and open fractures was 1.6% and 8.0%, respectively. These incidences are similar to those seen in the largest investigation of tibia shaft fractures in developed countries, which were 1.9% in closed fractures and 8.8% in open fractures [10]. This result is surprising, as many study participants had injuries resulting from motor vehicle accidents (71.4%), experienced long times to surgery (64 h for open and 76 for closed fractures), and did not have health insurance (<20% had insurance). The typical time between injury and surgery in developed countries for open fractures has been reported to be 9.8 h, which is drastically shorter than the mean time to surgery of 64 h for open fractures observed within this study [11]. Contributing factors may include patients living in rural areas of India unable to travel to a hospital in an appropriate time, as well as patient overcrowding in a hospital. Overcrowding can result in delayed treatment, long patient waiting time and stay, overburdened working staff, and poor patient outcomes [12]. Despite these factors, one possible explanation for the low incidence of infection seen in our study is that participants received prophylactic antibiotics for a mean of 8.3 days in closed fractures and 9.1 days in open fractures, which is much longer than North American standard practice [13–15]. The typical length of prophylactic antibiotic use for tibia fractures in the literature ranges from 1–5 days, demonstrating an extended length of prophylactic antibiotic use seen within our cohort [15, 16]. However, this is specific to open fractures in India without evidence of use in closed fractures. The widespread and prolonged use of prophylactic antibiotics within this study may be a contributor to the low incidence of infection given that prophylactic antibiotic use has been suggested to reduce the risk of infection after internal tibial fracture fixation by 29% [17].

Although the incidence of infection seen within our study is relatively low, infection management may not have been optimal. The large number of participants with an unresolved infection at 12 months post-fracture (10 open and 9 closed fractures) suggests that once an infection was present there was difficultly in managing it. Current guidelines outline drainage, debridement, and specific antibiotic prescription as the hallmark treatment regimen for SSI; however infection management within our study was seen to primarily consist of antibiotic alone (53.8%) [18, 19]. The prolonged length of infection duration within our study may be a result of the limited use of surgical intervention to address infection, as only 38.5% of infections were treated surgically despite surgical debridement being a core component of SSI treatment in current guideline recommendations [20].

Our EQ-5D results suggest that reducing the incidence of infection is important in increasing participant recovery and quality of life parameters following a tibia fracture, whether it be open or closed, as infection resulted in longer time required to heal and significantly decreased health related quality of life measures. These results align with the literature, which shows that the occurrence of infection significantly decreases participant quality of life when compared to individuals who avoid surgical site infection [21].

This study is strengthened by its prospective design. The study had clearly defined eligibility criteria prior to study initiation to ensure that all included participants were an appropriate representation of the target population. Additional strength was gained through the large sample size and use of multi-centre recruitment. The study also was able to capture details regarding current clinical practices in India, as standardized treatment methods and antibiotic regimens were not provided for the study. Attending surgeons treated patients as they would in typical clinical practice, and eligibility criteria did not remove patients based on clinical factors such as prolonged delay between injury and treatment. This was important to ensure that results were an honest depiction of tibial fracture patients seen in India. The study is limited by the low number of events seen within the cohort, as only 23 infections were seen across all participants. This may be a result of the large proportion of closed fractures within the study, as they are generally at low risk of infection. The low number of infections decreases the power of our statistical analysis, as the sample size of infections is small. Another potential limitation of this study is that the hospitals that participated may not be representative of the average Indian hospital, as they were large and modern facilities with experience in clinical research. An additional limitation arises due to the inclusion of all types of tibial fractures, making the results more difficult to be used for drawing conclusions for specific tibial fracture types. Furthermore, the study results are specific to India, and cannot appropriately be generalized to other patient populations. However, while these infection rates are specific to India, they align with previously published results of tibia fracture infection after internal fixation within LMICs [16]. Lastly, there exist several risk factors for surgical site infections that were not investigated outside of smoking and diabetes. Future studies should aim to explore other risk factors and determine if there is a relationship between incidences of infection and fracture type/location.

This is the first investigation to our knowledge to provide a thorough overview of the incidence of infection, prognostic factors, prophylactic antibiotic use, infection management options, and patient quality of life for patients who undergo internal fixation of a tibia fracture in India. This investigation allows for the calculation of required subjects for additional studies in this area of research. Although the incidence of infection within this study is similar to that observed in North America, participants in this study received prophylactic antibiotics for considerably longer than North American standard practice.

## Conclusion

The incidence of infection within this study is similar to that seen in developed countries within the current literature. The duration of prophylactic antibiotic use in India was much longer than typical North American regimens. Future research should aim to identify the best practices for management of infection and for prophylactic antibiotic use to ensure a strict treatment algorithm is established for the management of soft tissue and fracture morphology while avoiding unnecessary overuse.

## Abbreviations

ASA: Society of anesthesiologists
EQ-5D: EurQol-5 Dimensions
LMIC: Low and middle income countries
SSI: Surgical site infections
